# The Impact of the COVID-19 Pandemic on Childhood Obesity: A Review

**DOI:** 10.7759/cureus.45470

**Published:** 2023-09-18

**Authors:** Eftychia Ferentinou, Ioannis Koutelekos, Despoina Pappa, Panagiota Manthou, Chrysoula Dafogianni

**Affiliations:** 1 Nursing, Agia Sofia Children’s Hospital, Athens, GRC; 2 Nursing, University of West Attica, Athens, GRC; 3 Cath Lab, Erikos Ntinan Hospital, Athens, GRC; 4 Nursing, National and Kapodistrian University of Athens, Athens, GRC

**Keywords:** covid-19, obesity and nutrition, childhood, eating habits, school closure

## Abstract

The coronavirus disease 2019 (COVID-19) pandemic has changed many families’ eating habits and lifestyles. The main aim of this study was to investigate the association between COVID-19 and childhood obesity across the scientific literature. Literature reviews have shown that the current COVID-19 pandemic may play a major negative role in the global fight against childhood obesity. School closures, changes in routine, loss of structure, and loss of control were negatively associated with childhood obesity during the COVID-19 period. In addition, physical inactivity, irregular sleep, increased smartphone/TV screen time, and sedentary life may have played a significant negative role in social distress among children and adolescents. It has been argued that school closures during the pandemic have the potential to increase the prevalence of childhood obesity. Finally, family violence was predicted to increase during the pandemic, putting already vulnerable children at increased risk. The pandemic caused significant morbidity and mortality, straining healthcare systems, shutting down economies, and closing school districts. Pandemic future planning should involve stakeholders, including governments, schools, and families, who should make every effort to minimize the impact of the COVID-19 outbreak on childhood obesity.

## Introduction and background

The 2019 outbreak of coronavirus disease 2019 (COVID‐19), caused by severe acute respiratory syndrome coronavirus 2 (SARS‐CoV‐2) infection, spread rapidly around the world. By November 15, 2022, 632,533,408 confirmed cases of COVID-19, including 6,592,320 deaths, had been reported worldwide to the World Health Organization (WHO). As the severity of the disease in children was moderate, it was noted that the impact of COVID-19 on the pediatric population was extremely high. Many families that struggled with lockdown due to the COVID-19 pandemic were forced to make significant changes to lifestyle habits. Because of the lockdown, millions of people around the world had to deal with major economic turmoil that affected their livelihoods [1]. COVID-19 has played a negative role in the global fight against childhood obesity [2]. The short-term closure of educational institutions was challenging for children and had a negative impact on their physical and mental health destroying the sociability, communicativeness, and social interaction among students [3,4].

Loades et al. found a link between social anxiety and loneliness/social isolation in children and adolescents during the COVID-19 pandemic [5]. This review provided evidence that by fostering an unprecedented obesogenic environment, the COVID-19 pandemic aggravated the childhood obesity crisis and contributed to massive weight increases in children. In response to the COVID-19 outbreak, many countries adopted uncompromising measures, including closing schools and quarantines. In many countries, the epidemic of childhood obesity emerged as a significant public health issue [5].

Obesity in childhood increases the likelihood of obese adults, and adult obesity has been linked to a higher risk of morbidity [3]. As a result, combating the global epidemic of childhood obesity has been elevated to a top public health priority. Although the measures required to curtail the COVID-19 pandemic were frequently taken to achieve infection control, they could significantly harm the mental and physical health of children [3]. In this setting, the physical, dietary, and psychosocial elements that encourage childhood obesity worked in concert to create an environment particularly conducive to obesity. Children were exposed to new and unfamiliar stressors as a result of quarantine and freedom restrictions, which exacerbated the childhood obesity pandemic. Physical, nutritional, and psychosocial factors that promoted obesity in children in this particular situation contributed complementarily to an unprecedented obesogenic environment. Quarantine and restriction of freedom imposed new and unfamiliar stressors on children, exacerbating the childhood obesity epidemic [2]. The COVID-19 pandemic has led to significant health, economic, financial, and social consequences associated with the increasing number of reported individuals with eating disorders [6].

## Review

The relationship between COVID-19 and childhood obesity

**Figure 1:**
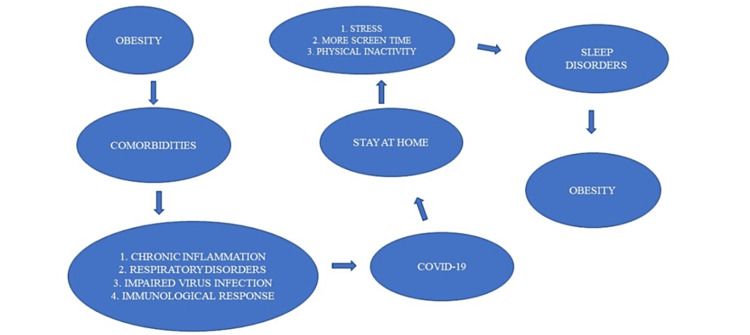
Interrelationships between obesity and COVID-19.

Over the past 40 years, there has been a noticeable increase in childhood obesity and overweight. By 2020, 39 million children under the age of five were predicted to be overweight or obese by the WHO. The prevalence of obesity among children and adolescents aged 5-19 increased from 1% in 1975 to 6% in girls and 8% in boys in 2016 [7]. Childhood quality of life can be negatively impacted by being overweight or obese, and these conditions also increase the probability of developing life-limiting comorbidities and being overweight as an adult. Therefore, allowing childhood obesity rates to increase can result in significant economic and health issues for future societies, not to mention increased susceptibility to pandemic outbreaks of viruses such as SARS-CoV-2. Type 2 diabetes, high blood pressure, heart disease, stroke, and even some types of cancer have been linked to childhood obesity [8]. Figure 1 illustrates the interrelationship between COVID-19 and childhood obesity. Because of the shame and isolation associated with their condition, obese children are more likely to experience sadness and eat more frequently under stress than their classmates who are of normal weight.

Before the COVID-19 pandemic, despite rising rates of severe obesity, the prevalence of obesity in children and adolescents had plateaued in many high-income countries. However, in low-income and middle-income countries, obesity prevalence had risen [9]. One of the main behavioral risk factors for childhood obesity is inactivity among children [10]. According to reports, the rate of childhood obesity in the United States may have increased by 0.85 percentage points per month over the summer of 2020 [11]. After six months of school cancellations, there ought to have been a 5.1% increase at this average rate [12]. The physical inactivity caused by the isolation and quarantine increased the average body mass index (BMI) by up to 0.198, with boys being slightly more affected [12]. Additionally, some families developed new interests or used new resources for physical exercise during this time, which could help explain why the projected growth is expected to be lower [11,12].

Besides, there was evidence to suggest that closing schools during a pandemic may increase the incidence of pediatric obesity [11,12]. Children with obesity found it difficult to maintain a healthy lifestyle throughout the COVID-19 quarantine, despite the fact that the pertinent group was a part of a hospital-based food education program [12]. It was well known that obese patients during this period experienced high levels of stress, which led them to live sedentary lifestyles and eat unhealthily [13]. The inability to observe the five meals and the incomplete noon meal, on the other hand, was possibly linked to the rise in feeling hungry and nibbling. These positive behaviors were not fully formed. These incorrect eating habits undoubtedly had a relationship with the hardships of this time, particularly the tension and anxiety caused by the abrupt and unanticipated changes in lifestyle. In fact, during the COVID-19 lockdown, adults’ perceptions of hunger at several times of the day (before, in between, and following dinner) increased as well [14].

Moreover, all children and adolescents treated in obesity outpatient and rehabilitation clinics during the pandemic had BMI-standard deviation scores (SDS) that were marginally higher than those from two years ago. It is feasible that more children with greater BMI-SDS and disease burdens visited the facilities. The BMI-SDS of the mainly normal-weight children and adolescents increased by 0.06 over the course of three months (which would translate to an increase in BMI-SDS of 0.24 over the course of 12 months), according to data from a pediatric cohort (primarily from eastern German states) [15]. According to Jia et al., the average BMI increased across all individuals. Following the lockdown [16], the total burden of obesity and overweight dramatically increased. Similar findings were seen in the Pellegrini et al. study, where it was shown that, on average, throughout the lockdown, self-reported weight and BMI significantly increased by 1.51 kg and 0.58 kg/m^2^, respectively. Kim et al. reported similar findings, with mean BMI rising from 26.7 to 27.7 kg/m^2^ across all individuals. Because there were fewer possibilities for physical activity during the COVID-19 pandemic, likely as a result of limitations, it appeared that it was challenging for obese children and adolescents to prevent gaining weight or to achieve weight loss. Weight gain might have also been influenced by alterations in eating behaviors, such as more frequent snacking at home.

Additionally, during the pandemic, less medical treatment was provided to children and their families [17]. A concerning rise in the percentage of children who were overweight or obese during the COVID-19 pandemic has also been shown, along with an increase in the standardized mean BMI scores in children aged 7-10 [17]. This outcome is comparable to prior research from several countries that noted an accelerated rise in BMI-SDS during the COVID-19 pandemic. The frequent closures of schools and athletic facilities were most likely to blame for the sharp rise in the proportion of children who were overweight or obese [18-20]. School closures, routine changes, loss of structure, and loss of control were negatively associated with childhood obesity during COVID-19 [21].

Changes to routine

Cooking appeared to be the best way to pass the time during lockdown. Nicodemo et al. emphasized that participants reported feeling hungrier on average, particularly in the afternoon, which was accompanied by an increase in the consumption of sweets and biscuits. Only 21.6% of the population exercised frequently at home during lockdown, while 51.1% of young individuals participated in food preparation, particularly making desserts (26.5%). The same study focused on water consumption, another eating behavior that children typically overlook. Only 46.6% of the population was found to routinely consume 1.5 L of water each day [22]. There are claims that children and adolescents with eating disorders saw a worsening of their symptoms, a rise in isolation, and a rise in hospital admissions as a result of the COVID-19 pandemic [15].

School closure

In addition to providing an opportunity for education, going to school gave children a good excuse to leave the house and explore the neighborhood’s open spaces. Schools not only provide pedagogy and academics but also a window of freedom, a chance for connection with peers and seniors, and psychological consolation. Personal cleanliness, exercise, healthy eating, and good body habits are all important concepts that schools help instill in students [23]. This was unquestionably true for a large number of kids and teens. There was a subset of kids and teens with social phobia for whom the school shutdown caused a temporary decrease in suffering and the lack of exposure to anxiety-provoking scenarios in the school setting. Although long-term school closures raised the risk of childhood obesity and other chronic diseases due to a positive energy balance, they deprived kids of the benefits of physical activity for their physical and mental health [12]. Additionally, a meta-analysis of 22 studies involving 143,603 kids showed that depression is more common among obese kids. Children who were obese had a 1.32 times higher risk of depression than children who were normal weight, and obese females in particular had a 1.44 times higher risk of depression than their normal-weight female classmates.

Clinicians should consider screening obese girls for depressive symptoms because the hazards for females continue into adulthood [24]. The use of digital tools such as social networking and video conferencing may have allowed some kids and teenagers to stay in touch with their peers. Nevertheless, socially anxious children and adolescents might have shied away from these approaches to communication or had trouble using digital means of exchange. For instance, seeing oneself on screen during a video conference may raise negative, self-focused attention, and frequent delays and interruptions in dialogue may be misinterpreted as others being unpleasant or indifferent [25,26]. Although temporary school closures brought on by medical and other crises are regrettably nothing new, the current educational disruption is unprecedented in its global scope and pace and, if it persists, could endanger the right to education. According to Rundle et al., increasing the amount of time spent outside of class alone could result in a significant rise in childhood obesity [27]. According to Rundle et al., the pandemic worsened food insecurity, increased dependency on processed foods, and decreased possibilities for outdoor exercise [27].

On the other hand, the pandemic may have consequences that lower the likelihood of a child being obese. In reaction to social distancing regulations, for instance, families could eat out less frequently [28,29]. Nevertheless, the severity of the pandemic may force authorities and educational institutions to take more decisive action than they would in previous disruptive situations. Childhood obesity and decreased cardiorespiratory fitness will be the outcomes of this extended period of inactivity, inconsistent sleep patterns, unfavorable dietary habits, sedentary lifestyle, and increased use of smartphones and televisions during lockdowns and school closures [3]. The psychological health of children and adolescents was harmed during the COVID-19 pandemic, and psychosomatic and psychological symptoms (such as anxiety symptoms and depression symptoms) occurred more frequently, according to surveys conducted in Germany [17,30]. Governments all over the world took action, mostly during the first semester of 2020 to suspend face-to-face instruction in schools to stop its growth, affecting almost 95% of all students worldwide, the biggest interruption to education in history [31].

## Conclusions

The COVID-19 pandemic had a significant impact on society and caused social and economic inequality to worsen. The pandemic exerted pressure on healthcare systems, shutting down economies, and closing school districts in addition to generating significant morbidity and mortality. Future pandemic preparation needs to take into account the scenario described in this analysis as it is of utmost public health relevance. This review highlights the increase in obesity rates and possible causes. To reduce the effects of the COVID-19 pandemic on childhood obesity, all relevant parties, including governments, institutions of higher learning, and families, must make every effort.
